# Patterns of Genetic Variation across Altitude in Three Plant Species of Semi-Dry Grasslands

**DOI:** 10.1371/journal.pone.0041608

**Published:** 2012-08-01

**Authors:** Thomas Hahn, Chris J. Kettle, Jaboury Ghazoul, Esther R. Frei, Philippe Matter, Andrea R. Pluess

**Affiliations:** Institute of Terrestrial Ecosystems, Ecosystem Management, ETH Zurich, Zurich, Switzerland; University of Konstanz, Germany

## Abstract

**Background:**

Environmental gradients caused by altitudinal gradients may affect genetic variation within and among plant populations and inbreeding within populations. Populations in the upper range periphery of a species may be important source populations for range shifts to higher altitude in response to climate change. In this study we investigate patterns of population genetic variation at upper peripheral and lower more central altitudes in three common plant species of semi-dry grasslands in montane landscapes.

**Methodology/Principal Findings:**

In *Briza media*, *Trifolium montanum* and *Ranunculus bulbosus* genetic diversity, inbreeding and genetic relatedness of individuals within populations and genetic differentiation among populations was characterized using AFLP markers. Populations were sampled in the Swiss Alps at 1800 (upper periphery of the study organisms) and at 1200 m a.s.l. Genetic diversity was not affected by altitude and only in *B. media* inbreeding was greater at higher altitudes. Genetic differentiation was slightly greater among populations at higher altitudes in *B. media* and individuals within populations were more related to each other compared to individuals in lower altitude populations. A similar but less strong pattern of differentiation and relatedness was observed in *T. montanum*, while in *R. bulbosus* there was no effect of altitude. Estimations of population size and isolation of populations were similar, both at higher and lower altitudes.

**Conclusions/Significance:**

Our results suggest that altitude does not affect genetic diversity in the grassland species under study. Genetic differentiation of populations increased only slightly at higher elevation, probably due to extensive (historic) gene flow among altitudes. Potentially pre-adapted genes might therefore spread easily across altitudes. Our study indicates that populations at the upper periphery are not genetically depauperate or isolated and thus may be important source populations for migration under climate change.

## Introduction

Altitudinal gradients comprise an assemblage of environmental, especially climatic, variables which influence the distribution of plant species [1] and, potentially, population genetic variation [2]. Climate change may thus alter species distributions in mountain regions. Locally, species might become extinct [3], [4]. Recent studies also indicate an upward migration of plant species along altitudinal gradients [5]–[7]. Within species, pre-adapted genes for lower altitudinal climatic conditions might move into upper peripheral populations. Due to the uncertainty of plant responses to climate change in mountain regions, an understanding of current distribution patterns of population genetic diversity and differentiation [8] along altitudinal gradients is fundamental for establishing management strategies for resilient species and their habitats.

The potential for altitudinal climatic gradients to influence the distribution of genetic variation within and among plant populations is highly complex and variable among species [2], [9]. Generally, decreasing temperatures associated with increasing altitudes cause a decline in resource availability [6]. Habitat suitability thus decreases at the upper edge of the altitudinal range where population size might decline. One potential theoretical consequence of this is smaller effective population sizes (*N*_e_) in upper peripheral populations compared with lower altitude populations closer to the range centre, a so-called Abundant Centre distribution [2], [10]. In small populations genetic diversity is often reduced and inbreeding may increase [11], [12]. In self-compatible, predominantly outcrossing species, low genetic diversity and high inbreeding potentially cause loss of fitness due to fixation of deleterious alleles and inbreeding depression [12]–[14] which could affect future population persistence. Similarly, biparental inbreeding in self-incompatible plants can lead to increased inbreeding depression [15], whereas increased self-compatibility can cause reduced genetic load due to purging of deleterious alleles and thus less severe inbreeding depression [16].

While population size is a major determinant of genetic variation in populations with Abundant Centre distribution, similar genetic patterns might arise independent of population size. For example, upper peripheral populations could show reduced genetic diversity, increased inbreeding and genetic differentiation compared to lower more central populations, because of limited pollen or seed mediated gene flow from higher altitudes [17] and higher selection pressure under harsher climatic conditions. Steep climatic gradients within mountain ranges could reduce gene flow across altitudes due to asynchronous flowering phenology [18] or disjunct pollinator communities [19]. Reproductive isolation might increase with altitude leading to reduced genetic connectivity among populations at different altitudes and/or among populations at the same altitudinal level on different mountains. These processes might be enhanced in small populations within the upper periphery of an Abundant Centre distribution.

Species characteristics, especially the pollen dispersal mode, influence patterns of gene flow and consequently patterns of genetic variation in alpine environments [20]. Wind pollinated species may have longer distance pollen dispersal than insect pollinated species under certain conditions. For example, pollination success of wind pollinated species might be more stable at high altitudes than insect pollination, where pollinator activity could be reduced due to the colder conditions [18], [21]. It is thus conceivable that population isolation is less pronounced among altitudes in wind-pollinated species, which could lead to differences in species responses to changing climate as a function of pollen dispersal mode.

The species rich semi-dry grassland is an important and relatively common habitat type along altitudinal gradients in the European Alps [22]. Within the last 60 years about 90% of the total area of semi-dry/dry grasslands has been lost in Switzerland due to land use change [23]. Abandonment of continuous extensive management, land use intensification and urban sprawl in concert with climate change are expected to cause further habitat reduction and fragmentation with the potential to severely threaten this valuable ecosystem [6], [23]. Semi-dry grasslands are highly fragmented across the lowlands of Switzerland, and much of lowland Europe, while larger areas in the Central Alps are still present from montane to just alpine levels [24]. Understanding the patterns of genetic variation within characteristic species of these grasslands is necessary to support effective management of this habitat and to ensure persistence and adaptability of its species in the face of anthropogenic climate change.

At montane and submontane altitudes, semi-dry grassland patches are often separated by areas of forest which protect the area below them from avalanches, landslides and rock falls (so called ‘protection forests’). These protection forests have increased in size over recent decades [25]. Such human modification to the spatial arrangement of landscape elements [26] may increase fragmentation of semi-dry grassland areas and might change species distribution patterns from those predicted under the Abundant Centre hypothesis along altitudinal gradients. Land-use change and its implications for population sizes and densities are often neglected in population genetic studies along central-peripheral gradients [27], but see [28].

In this paper we investigate differential patterns of genetic diversity, inbreeding and population genetic structure in three common plant species of semi-dry grasslands in population pairs at 1200 and 1800 m a.s.l. in the Swiss Alps using the amplified fragment length polymorphism (AFLP) technique. Our study species were the wind-pollinated grass *Briza media* and the two insect-pollinated herbs *Trifolium montanum* and *Ranunculus bulbosus*. Anthropogenic land cover change may undermine putative Abundant Centre demographic patterns, as outlined above. Therefore, our expectation was that population size does not follow an Abundant Centre distribution pattern along our studied altitudinal gradients. To test this expectation we estimated population size and geographic isolation.

Specifically, we investigated patterns of population genetic diversity in the context of the altitudinal gradient and pollen dispersal mode using the following hypotheses as a heuristic framework: (i) Neutral genetic diversity is lower and inbreeding is higher in upper peripheral populations compared with populations at lower elevations. (ii) Upper peripheral populations are more differentiated and have increased individual relatedness within populations compared to populations at lower elevation. (iii) Altitude has a smaller effect on the above mentioned population genetic traits in the wind-pollinated species compared to the insect-pollinated species.

## Results

### Evaluation of Abundant Centre Pattern and Evidence for Climatic Differences at the Two Altitudes

Estimators for population size (i.e. individual density, inhabited area within circles of 100, 200 and 400 m radii around the centroid of the sampled individuals within each population) and distance to nearest neighbouring habitat patch did not differ among the two altitudinal levels in pair-wise Wilcoxon tests (within species Bonferroni corrected *P*- values (*P*_Bonf_) of >0.05; estimates in Table 1), despite of *B. media* which tended to have larger inhabited semi-dry grassland patches within 400 m radius at 1800 compared with 1200 m a.s.l. (*P*_Bonf_ = 0.081). Average July temperatures were, for all populations, appr. 4°C lower at 1800 than at 1200 m a.s.l.

**Table 1:** Appr. altitude of population (m a.s.l.), number of sampled populations (n), average individual density category 1 (appr. 1–2 per****25****m^2^), 2 (appr. 3–15 per****25****m^2^) and 3 (appr. >15 per****25****m^2^); distance to nearest neighbouring population and inhabited semi-dry grassland patch area within three different radii around sampling centres in *Briza media*, *Trifolium montanum* and *Ranunculus bulbosus*.

Species	m a.s.l.	n	Individual density	Nearest neighbour distance [m]	Patch area100m [m^2^]	Patch area200m [m^2^]	Patch area400m [m^2^]
*Briza media*	1200	10	2.2±0.2	392±145	12‘955±1‘948	31‘104±7‘367	51‘075±12‘794
	1800	10	2.1±0.2	409±247	18‘417±2‘374	45‘860±9‘106	112‘248±23‘169
*Trifolium montanum*	1200	10	2.0±0.2	411±149	13‘741±1‘827	29‘532±6‘232	53‘910±12‘409
	1800	10	1.8±0.1	124±320	19‘043±1‘719	49‘466±7‘739	102‘859±22‘752
*Ranunculus bulbosus*	1200	9	1.7±0.1	180±770	10‘883±1‘276	25‘098±5‘181	47‘055±11‘850
	1800	9	1.6±0.2	271±101	13‘062±3‘023	33‘149±9‘798	55‘462±15‘082

Values are averaged over populations within altitude (±SE).

### Effect of Altitude on Genetic Diversity & Inbreeding within Populations

The three genetic diversity measures, expected heterozygosity (*H*_e_), percentage of polymorphic loci (PPL) and Bayesian cluster diversity (BCD) did not differ between low (1200 m a.s.l.) and high altitude populations (1800 m a.s.l.) in all three species (*P*>0.098 for all comparisons; Table 2). The estimated inbreeding coefficient (*f*_AFLP_) did not differ among altitudes in *T. montanum* and *R. bulbosus* (*P*>0.130), while in *B. media* high altitude populations showed larger *f*_AFLP_-values compared to low altitude populations (*P* = 0.020). This difference became marginally significant (*P* = 0.064) after setting the *f*_AFLP_ -values with low confidence to “0” (i.e. in 13 out of 20 cases the estimates did not significantly differ from 0).

**Table 2:** Appr. altitude of populations (m a.s.l.), number of sampled populations (n), mean (± SE) of expected heterozygosity (*H*_e_), percentage of polymorphic markers (PPL), Bayesian cluster diversity (BCD), average pairwise *F*_ST.overall,_ and average relatedness coefficient (rc), inbreeding estimate (*f*_AFLP_) among low (1200 m a.s.l.) and high altitude (1800 m a.s.l.) populations in the three study species *Briza media*, *Trifolium montanum* and *Ranunculus bulbosus*.

Species	m a.s.l.	n	H_e_	*P*	PPL	*P*	BCD	*P*	*f*_AFLP_	*P*	*F*_ST.overall_	*P*	rc	*P*
***Briza media***	1200	10	0.264±0.004	0.160	85.79±0.707	0.490	0.591±0.039	0.160	0.089±0.049	0.063	0.092±0.006	0.001	0.143±0.020	0.004
	1800	10	0.259±0.004		80.91±3.025		0.537±0.027		0.143±0.051		0.105±0.005		0.205±0.017	
***Trifolium montanum***	1200	10	0.235±0.004	0.846	58.29±3.436	0.940	0.591±0.039	0.431	0.093±0.038	0.220	0.108±0.006	0.083	0.190±0.034	0.064
	1800	10	0.235±0.006		58.08±3.954		0.537±0.027		0.141±0.056		0.126±0.010		0.259±0.042	
***Ranunculus bulbosus***	1200	9	0.167±0.005	0.734	53.96±1.897	0.470	0.591±0.039	0.820	0.051±0.058	0.380	0.070±0.003	1.000	0.127±0.012	0.570
	1800	9	0.170±0.004		55.64±1.578		0.537±0.027		0.168±0.080		0.071±0.004		0.126±0.014	

*P*-values (*P*) of two-sided pairwise exact Wilcoxon tests of low vs. high altitude populations are presented. *f*_AFLP_ averages include all estimates independent of their confidence.

### Genetic Differentiation Among Populations

**Figure 1:**
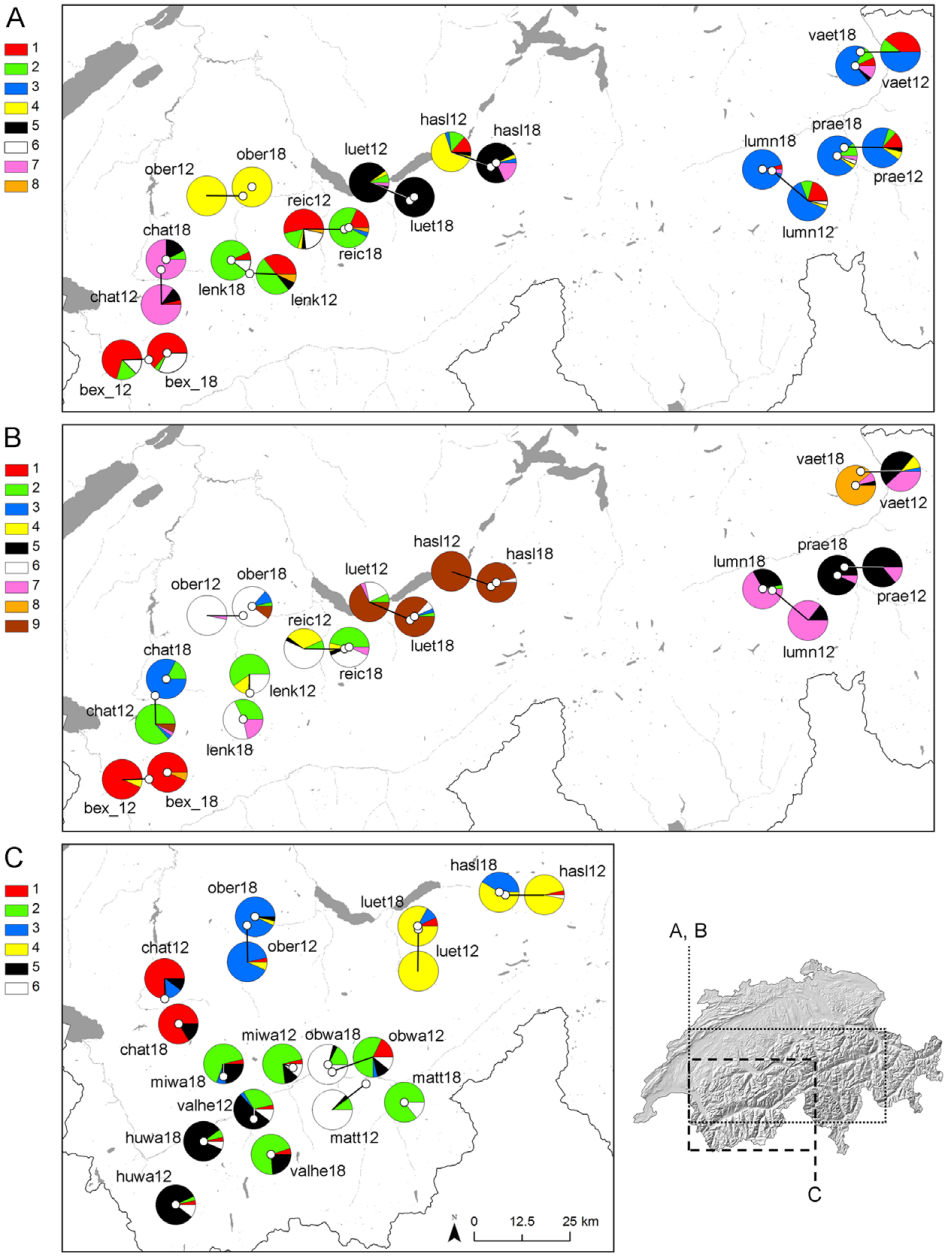
Geographic distribution of fractions of Bayesian clusters (encoded by different colours) per sampling area (white dots) in A) *Briza media*, B) *Trifolium montanum* and C) *Ranunculus bulbosus*. Details on sampling areas are provided in Table S1.

Overall average pairwise *F*_ST.overall_ values (± SE) were 0.099 (±0.004), 0.118 (±0.006) and 0.071 (±0.003) for *B. media*, *T. montanum* and *R. bulbosus*, respectively. All pairwise *F*_ST.overall_ values differed significantly from zero except the “Prae12” and “Prae18” pair in *T. montanum*. We evaluated four aspects of population differentiation at the individual and population level, using frequentistic and Bayesian statistics: (i) A Mantel test indicated isolation by distance relationships (*P*<0.001) with Mantel-r of 0.36, 0.50 and 0.55 in *B. media*, *T. montanum* and *R. bulbosus*. (ii) A hierarchical AMOVA showed that in *B. media*, *T. montanum* and *R. bulbosus* 8.1%, 12.0% and 7.7% of the variation, respectively, was explained by differences among locations (we defined locations as the sampling localities of a lower altitude population and a close by high altitude population), while 5.9%, 5.4% and 4.3%, respectively, was explained by differences within location (i.e. between the two altitudes). The rest was explained by differences within populations (>83%). (iii) A Principal Coordinates Analyses (PCoA) based on Nei’s standard genetic distances separated distinct grouping concurrent with the geographic distribution of the populations (Fig. S1). The PCoA distance between populations within a location was often larger than the PCoA distance to the closest population belonging to a different location. (iv) We estimated the numbers of Bayesian clusters per species in BAPS [29] without inclusion of geographic coordinates. In *B. media*, *T. montanum* and *R. bulbosus* eight, nine and six clusters were estimated respectively. In concordance with the IBD relationship and PCoA results, in each species Bayesian clusters mainly represented groups of neighboring populations (Fig. 1). In general the number and distribution of clusters showed no clear effect of altitude. Only in *T. montanum* the rare cluster No. 3 dominated the high altitude population “chat18” and the rare cluster No. 8 was most abundant in high altitude population “vaet18”.

**Figure 2:**
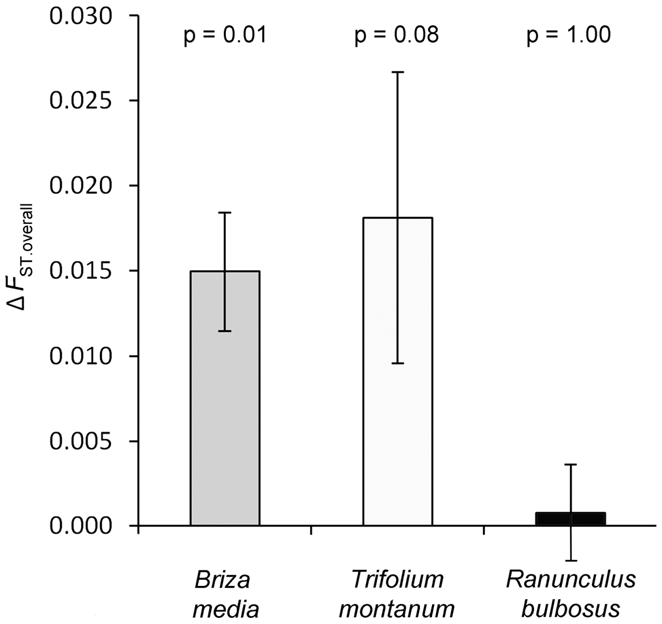
Difference in genetic differentiation (Δ average pairwise *F*_ST.overall_ ± SE) between high (1800 m a.s.l.) and low (1200 m a.s.l.) altitude populations in the three grassland species: *Briza media*, *Trifolium montanum* and *Ranunculus bulbosus*.

**Figure 3:**
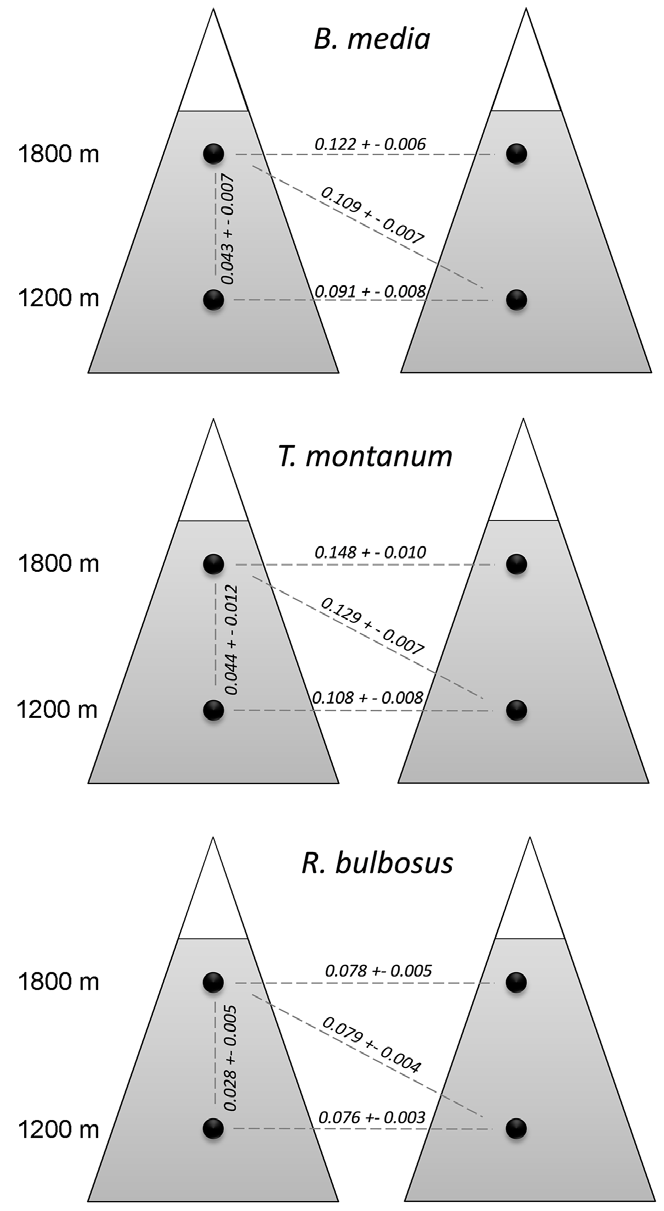
Average pairwise genetic differentiation (*F*_ST_ ± SE) among low (1200 m a.s.l.) and high altitude (1800 m a.s.l) populations in *Briza media*, *Trifolium montanum* and *Ranunculus bulbosus* among high altitude populations, among low altitude populations, between altitudes within one location and between altitudes in different locations.

Genetic differentiation of high and low altitude populations was summarized in two ways. First, differentiation of a given population to all other populations (i.e. *F*_ST.overall_) and to the populations within the same altitude (i.e. *F*_ST.alt_) was calculated. In *B. media* high altitude populations were more differentiated than low altitude populations (*F*_ST.overall_: *P* = 0.010, *F*_ST.alt_: *P* = 0.002, respectively). This was similar in *T. montanum*, however overall differentiation was only marginal (*F*_ST.overall_: *P* = 0.084, *F*_ST.alt_: *P* = 0.002, respectively). In *R. bulbosus* genetic differentiation was equal between altitudes (*F*_ST.overall_: *P* = 1.000, *F*_ST.alt_: *P* = 0.910, respectively) (Fig. 2). Second, we calculated population differentiation between high and low altitudes within and between locations (Fig. 3). Based on the comparison of 95% confidence intervals, average pairwise *F*_ST.overall_ estimates among populations could be ranked for *B. media* and *T. montanum* as follows: among high altitude populations > among low altitude populations > between high and low altitude populations within the same location. *F*_ST_ between high and low altitude populations belonging to different locations (i.e. the population within the same pair was excluded) was similar to the estimates among high as well as among low altitude populations. In *R. bulbosus F*_ST_ estimates among high and low altitude populations within the same location differed from the other three groups of pair-wise *F*_ST_-values.

### Genetic Relatedness within Populations

At the within population level, the average relatedness coefficient (rc) was greater in high compared to low altitude populations in *B. media* (*P* = 0.004). In *T. montanum* rc tended to be larger at high altitude (*P* = 0.064) and there was no difference among altitudes in *R. bulbosus* (*P* = 0.570, Table 2). In *T. montanum* the average rc increased with increasing *f*_AFLP_ (*P* = 0.010; 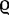 = 0.56). In contrast, no significant relationships among these two variables were found in *B. media* and *R. bulbosus* (*P* = 0.330; 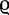 = 0.23, *P* = 0.190; 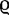 = −0.33, respectively).

## Discussion

### Distribution of Populations

Our population size estimates suggest that populations at high altitudes, which are in the upper range periphery, are of similar size and have similar plant densities as those at lower altitudes closer to the range centre. The total area of semi-dry grassland between 1700 and 1900 m a.s.l. is larger (53****km^2^) than between 1100 and 1300 m a.s.l. (31****km^2^) in Switzerland [24] (Fig. S2). One potential reason for this distribution pattern could be the higher forest cover at 1200 compared with 1800 m a.s.l. [25], driven by the establishment and conservation of protection forests. Moreover, at lower altitudes agricultural intensification reduced the area of semi-dry grassland over recent decades [23]. Contrary to our study, population sizes of the subalpine shrub *Lavandula latifolia* declined from low central to high peripheral altitudes, following an Abundant Centre pattern [10]. A meta-analysis with studies on species ranges [30] showed that of 145 tests for Abundant Centre patterns involving 121 species, only 39% affirmed their occurrence, demonstrating that the generality of the Abundant Centre Hypothesis is low.

### Genetic Variation and Inbreeding at Contrasting Altitudes

Populations from low and high altitudes of the three species under study generally did not differ in genetic diversity and inbreeding estimates. These findings suggest that the altitudinal gradient is not a major driver of population genetic diversity in these grassland species, despite the considerable difference in July temperature (on average 4°C) between the two altitudes.

One interpretation of the absence of any altitudinal effect on genetic diversity is that there is no Abundant Centre effect. This could be explained by anthropogenic drivers of habitat distribution and patch sizes such as reforestation and agricultural intensification. These processes can have a major impact on population sizes and thus intraspecific genetic diversity in pre-alpine grasslands, potentially counteracting effects of natural gradients on population genetic diversity. The similarly large population sizes at both altitudes appear to contain relatively large gene pools. Indeed, the *H*_e_-values in our study were in the medium range found in AFLP studies of herbal species [31]. Generally, high genetic diversity can be maintained in large gene pools because genetic drift is low. The occurrences of large gene pools, across the entire species ranges, are thought to have resulted in equal levels of genetic diversity in latitudinal central and peripheral populations in other herbaceous plant species such as *Stipa capillata* [32] and *Vaccinium stamineum* [33].

The rate of gene flow among populations at different altitudes could mitigate the potential effects of altitude on genetic diversity. Particularly, pollen flow [34] may be high among large populations which produce large volumes of pollen [35]. The local management strategy might also influence seed flow among populations (see below). Even low rates of genetic exchange among populations can be sufficient to maintain polymorphism [36]. For example, effective pollen flow among altitudes explained high levels of genetic diversity in the alpine species *Campanula rotundifolia* [37] and *Arabis serrata* [38].

In *B. media* high altitude populations were slightly more inbred compared to low populations. This is somewhat counterintuitive, since one would expect that increased inbreeding leads to a reduction in genetic diversity [39] which was not the case here. In *T. montanum* and *R. bulbosus* inbreeding did not differ among altitudes, which is similar to findings for the alpine grass *Poa hiemata* [9] and the subalpine forb *Rutidosis leiolepis* [14]. Low inbreeding levels might be due to relatively large gene pools at both altitudinal levels [9], [14] or due to a stable self-incompatibility system along altitudinal gradients. Independent of altitude, inbreeding seems to be related to the small-scale genetic structure in *T. montanum*, as indicated by its overall correlation with the average relatedness per population. Populations with higher individual densities tended to be more inbred (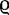 = 0.430, *P* = 0.061). The limited seed dispersal by gravity may lead to the aggregation of related individuals at short distances. This coupled with frequent pollen dispersal over short distances could increase inbreeding values.

Population genetic differentiation at high altitude was slightly more pronounced than at low altitude in *B. media* and *T. montanum*. Likewise, relatedness within populations was increased at higher elevation in these two species. Furthermore the two high altitude populations “vaet_18” and “chat_18” in *T. montanum* nearly exclusively consisted of individuals belonging to one of two rare Bayesian clusters each (Fig. 1), likely due to increased genetic isolation. Increased population differentiation at higher altitudes was also observed in *Poa hiemata* [9], *Rutidosis leiolepis* [14] and *Lavandula latifolia* [10]. Gene flow among populations seems to be reduced at higher altitudes. This may be explained by several alternative processes. The greater differentiation among populations from higher altitude might be due to the higher effective distance among high altitude populations which needs to be covered by gene flow. Gene flow among different mountains could follow a stepping stone pattern and actually may be hindered by at least two altitudinal transects, which pollen or seeds have to pass. It is as well possible that in upper peripheral populations reproductive isolation is increased due to the lack of genetic inflow from higher altitudes. In *T. montanum* higher differentiation could also be caused by decreased pollinator activity and lower pollination success at higher altitudes, as found in *Cytisus scoparius* [40]. For the clonal species *B. media,* increased resource allocation to clonal reproduction at higher altitudes [41] could have led to larger differentiation in populations at these altitudes [14] but see [42]. However, a greater allocation to clonal growth with altitude should also affect genetic diversity patterns, which was not the case in our study. Alternatively, asynchronous flowering phenology along the altitudinal gradient [43], [44] would restrict pollen flow between altitudes leading to more differentiation among high altitude and low altitude populations [9].

We hypothesized that altitude related effects would have lower influence on wind-pollinated compared with insect-pollinated species due to possibly higher pollen dispersal potential. The genetic patterns in *B. media* and *T. montanum* were however similar. This could be due to insects providing more targeted pollination over larger distances compared to wind pollination which can lead to pollen dilution over larger areas [45]. Indeed, below tree line filtration of aerial pollen by forest cover has been observed in some wind pollinated species [46]. In summary, we have little evidence to support the view that gene flow in wind-pollinated species is greater than in insect-pollinated species.

Contrary to *B. media* and *T. montanum*, population differentiation in *R. bulbosus* was similar at the two altitudes under study. The lower overall *F*_ST_ suggests more gene flow in *R. bulbosus* compared with the other two species. The geographic area sampled was slightly smaller in this species, which might partly explain the species difference [47]. However the scatter of the pairwise *F*_ST_-values, which is less affected by sampling design, appeared to be smaller in *R. bulbosus* than in the other two species (Fig. S3). The generalist flower morphology of *R. bulbosus* could allow for more extensive pollen flow among populations reducing the effect of altitude on genetic differentiation even further.

### Implications in the Face of Climate Change

Under future scenarios of climate change, sufficient genetic diversity in leading edge populations might be particularly important for successful upward migration as colonization events are often followed by genetic bottlenecks and decreasing population genetic diversity [48]–[50], but see [51]. Our results demonstrate that populations of three semi-dry grassland species are not genetically depleted in their current upper periphery. The three species also showed little differentiation among altitudes, perhaps due to relatively high connectivity caused by pollen movement as well as potential seed dispersal. Seed dispersal may even be facilitated by local management regimes [52], [53]. For example, although the seeds of the three species have no morphological adaptation for zoochory, they may be dispersed by livestock [52], which move among altitudes. Moreover, transportation of hay bales from alpine meadows to the valley bottom following harvest may ensure extensive seed dispersal. The intermediate diversity and high connectivity of semi-dry grassland plant populations are probably good prerequisites for future upward migration. The low neutral differentiation found in our study indicates that putatively locally adapted genes for e.g. warmer temperatures at lower altitudes might spread easily across the landscape.

Land-use changes especially abandonment of management could affect population stability in species rich grassland habitats. In *T. montanum* light competition in unmanaged grassland patches can lead to decreased survival of juveniles [54], indicating that land use abandonment may affect population persistence. Compared with common semi-dry grassland species, rare species with small population sizes and a limited geographic range, might show rather different patterns of genetic variation along the altitudinal gradient, which could cause increased vulnerability to climate change. However, for both common and rare species it is likely that a functioning habitat network will be necessary to maintain viable populations in the face of climate change [55], [56].

## Materials and Methods

### Ethic Statement

All necessary permits were obtained for the sampling of the leaf material. The permits were issued by the managers of the respective semi-dry grasslands.

### Model Organisms

*Briza media* (Poaceae), *Trifolium montanum* (Fabaceae) and *Ranunculus bulbosus* (Ranunculaceae) are mainly found in *Mesobromion erecti* grasslands below timber line and rely on continuous management [57]. *Briza media* as well tolerates more moist and acidic situations [58] and *T. montanum* also occurs at forest edges [59]. The three species are perennial and flower from appr. May to July [60]–[62] with a two to three weeks earlier onset of flowering in *R. bulbosus* (personal observation). All species are predominantly outcrossing [54], [61], [63]. *Briza media* is wind-pollinated, but also reproduces clonally [64], *R. bulbosus* is pollinated by generalist insects [61], [65] and *T. montanum* is predominantly pollinated by bumblebees and bees [54], [59]. The species are mainly bariochorous, thus seed dispersal is mostly restricted to short distances [54], [61], [66].

### Plant Material

The Swiss inventory of semi-dry and dry grassland (referred to as semi-dry grassland throughout the manuscript) areas of national conservational importance [24] was used to determine sampling locations of the three study species in the Swiss Alps. A paired approach was taken to sample leaf material at two altitudinal levels. The higher sampling level represents the species distributional upper periphery (appr. 1800 m a.s.l.; high) while the lower level lies closer to the range centre (appr. 1200 m a.s.l.; low) [62]. Average (± SE) altitudinal levels of the populations were for *B. media*, *T. montanum* and *R. bulbosus* 1194±15 and 1781±13 m a.s.l.; 1190±13 and 1786±15 m a.s.l.; 1196±22 and 1761±20 m a.s.l respectively. For *B. media* and *T. montanum* these paired samples were collected from ten locations and for *R. bulbosus* from nine locations (see Fig. 1). We endeavored to sample all species in the same locations, but *R. bulbosus* was not present at 1800 m a.s.l. in some locations of the other two species. We therefore also sampled *R. bulbosus* in south-western Switzerland (Valais) (Fig. 1). In *B. media* and *T. montanum* distances among populations within a sample location were one to five km, and the maximal distance among sample locations was 197 km. In *R. bulbosus* distances within sampling locations were one to 18 km and maximal distance among locations was 115 km. At each population leaf material was collected from 30 individuals with distances among individuals of at least four meters. Coordinates of sampled individuals were recorded with a GPS (Garmin 60CSx) and sampled material immediately dried with silica gel.

### Test for Abundant Centre Pattern and Evidence of Climatic Differences at the Two Altitudes Under Study

We characterized size and isolation of populations by estimating plant density as well as area and spatial isolation of inhabited semi-dry grassland patches. First, we estimated densities of flowering plants in the field at three density levels: 1 (appr. 1–2 per****25****m^2^), 2 (appr. 3–15 per****25****m^2^) and 3 (appr. >15 per****25****m^2^). Second, area of inhabited grassland patch was calculated for each population (Arc GIS 9.3.1) relying on a GIS vector model of dry grassland patches based on the TWW-inventory [24]. Due to the relatively high abundances of the focal species (personal observation) and the unknown spatial area of breeding units, we chose to measure inhabited patches of radii of 100, 200 and 400 m around the centre of sampled plants. These radii were chosen because the average maximum distance between samples within each population was appr. 200 m and minimal distances between two populations within a location were appr. 800 m. Third, we calculated patch-isolation via the nearest neighbor distance from the midpoint of all sampled individuals within a population to the most adjacent dry grassland patch inhabited by the respective study species.

In order to test for temperature differences in high (1800 m a.s.l.) compared to low altitude populations (1200 m a.s.l.) we determined average July temperatures based on temperature records from nearby climate stations (period 1961 to 1990, www.meteoschweiz.admin.ch) and interpolated across altitude with Daymet [67].

### AFLP Analyses

At least twenty AFLP primer combinations were screened per species for highest number of polymorphic loci and highest reproducibility of AFLP fragments. In each species four primer combinations were chosen to characterize genetic variation. The fluorescence labelled (Applied Biosystems, Foster City, California, USA) EcoRI- and non-labelled MseI-primers were for *B. media*: AGG (FAM)/CTAG; ACA (VIC)/CATG; AAC (NED)/CTGA; ATG (PET)/CTAG, for *T. montanum*: AGG (FAM)/CTA; ACC (VIC)/CTA; AAC (NED)/CAA; ATG (PET)/CTG and for *R. bulbosus*: AGG (FAM)/CGTA; ACC (VIC)/CAGT; AAG (NED)/CTAG; ATG (PET)/CTAG. AFLP analyses followed a modified version of the AFLP plant mapping protocol of Vos et al. [68] (for details see Text S1). Fragment analyses were performed on an automated capillary sequencer (ABI Genetic Analyser 3730, Applied Biosystems). Only individuals with loci derived of all AFLP primer combinations were included in the final analyses (*N* = 565, 578 and 512 for *B. media*, *T. montanum* and *R. bulbosus* respectively, Table S1).

Peaks of AFLP fragments between 50 and 500 bp were binned in Genemapper 3.7 (ABI) and peak heights were exported from un-normalized electropherograms for further semi-automatic genotyping with AFLPScore 1.4 [69]. To reduce genotyping errors, only loci with normalized average peak heights above 400, 300 and 300 relative fluorescence units were selected for genotyping *B. media*, *T. montanum* and *R. bulbosus*, respectively. In order to diminish bias in estimation of population genetic parameters, AFLP datasets were pruned from loci with allele frequency of >1– (3/*N*), where *N* is the total number of sampled individuals [70]. The genotyping process resulted in datasets with 121, 99 and 126 polymorphic loci for *B. media*, *T. montanum* and *R. bulbosus* (mismatch error rates 4.4%, 2.7% and 4.7%, respectively).

### Statistical Analyses of the Genetic Data

Initially the three datasets were scanned for AFLP loci under differential selection with outlier loci analysis in BayeScan 2.01 [71]. No markers were found to be linked to altitude (false positive rates were >5%, with posterior probabilities <0.79 and Bayes Factors <3), thus all loci were kept for following analyses. To test for sampling of different genets in *B. media* an identity analysis was carried out in Cervus 3.0.3 [72]. Due to the AFLP mismatch error rate of 4.4% in the AFLP dataset containing 121 loci, samples were still considered to belong to the same genet, if up to five loci were not matching in the comparison. The identity analysis in *B. media* showed that all samples did belong to different genets.

To assess neutral genetic diversity of populations we estimated expected heterozygosity (*H*_e_) with AFLP-SURV 1.0 [73] and percentage of polymorphic loci (PPL) with GenAlEx 6.4 [74]. *H*_e_ was calculated based on allelic frequencies following Zhivotovsky [75] taking an estimation of the inbreeding coefficient (*f*_AFLP_) into account (see below) [76]. As an estimate for the diversity and evenness of gene pools per population we used the analysis output from BAPS 5.3 [29], containing information on the assignment of individuals to Bayesian clusters, to calculate Simpson’s Diversity Indices for each population as implemented in the R-package Vegan. This index was termed “Bayesian cluster diversity” (BCD).

For each species, estimations of F-values (*f*_AFLP_) were obtained on an individual basis from FAFLPcalc [76] and averaged across populations for further analyses. We further tested, if *f*_AFLP_-values differed from zero by calculating bonferroni corrected 95% confidence intervals (CI) on the population level. If CIs included zero, the respective *f*_AFLP_ -estimate was set to zero for further analyses.

As measure for overall genetic differentiation pairwise *F*_ST_-values among populations were estimated as well as their corresponding 95% CI based on 1000 distance matrices bootstrapped over loci. For each population the pairwise *F*_ST_-values between the focal population and all remaining populations were averaged providing a point estimate for population genetic differentiation (*F*_ST.overall_) [9], [33], [77]. To assess genetic differentiation within the same altitudinal level (1200 and 1800 m a.s.l.) pairwise *F*_ST_-values between populations of the respective altitude were averaged (*F*_ST.alt_).

We further used four approaches for describing population differentiation:

We tested for spatial limitation of gene flow resulting in an isolation by distance pattern and carried out a Mantel-Test [78] as implemented in the software IBD Web Service 3.16 [79] including pairwise *F*_ST.overall_ values and log-transformed geographic distances. Significance was estimated via 10,000 permutations. (ii) Partitioning of genetic variation among locations, among altitudes and within populations was determined with a hierarchical analysis of molecular variance (AMOVA) using GenAlex and the Bayesian cluster analyses using BAPS. For the AMOVA the populations were grouped by locations and 999 permutations were done for significance testing. (iii) A multivariate principle coordinates analysis (PCoA) based on pairwise Nei’s standard genetic distances [80] among populations was used to determine the effects of geographic and altitudinal distances on genetic differentiation of population. (iv) Bayesian cluster analysis was done with the mixture model (without subsequent admixture modeling) using K-max of one to 20, three times each and without inclusion of geographic coordinates.

We further assessed the relatedness within populations by calculating Hardy’s among-individual relatedness coefficient (rc) in SPAGeDi 1.3 [81] which was then averaged per population.

To assess the differences between low and high altitude populations in the measures described above, Wilcoxon tests were carried out accounting for the paired sampling of the populations (i.e. paired tests). To evaluate the impact of inbreeding within populations on the genetic similarity within populations Spearman rank correlation test was done between *f*_AFLP_ and rc. Bonferroni corrections were applied whenever multiple tests were performed. If not otherwise stated, the analyses were carried out in R [82].

## Supporting Information

Figure S1 **PCoA plots of Nei’s Distances among populations in a)** ***Briza media*** **, b)** ***Trifolium montanum*** **and c)** ***Ranunculus bulbosus*** **.** Populations within the same location are connected by dashed lines.
(TIF)

Figure S2 **Estimates of total area [m^2^] of semi dry grasslands between 100 and 2000 m a.s.l. in Switzerland, based on the Swiss national inventory of dry grasslands (TWW).**
(TIF)

Figure S3 **Isolation by distance relationships between pairwise genetic (** ***F*** **_ST_) and geographic distances among populations at the same altitudinal level (red = at 1200 m a.s.l.; blue = at 1800 m a.s.l.) and among different altitudinal levels (green = among 1200 and 1800 m a.s.l.) in A)** ***Briza media*** **, B)** ***Trifolium montanum*** **and C)** ***Ranunculus bulbosus*** **.**
(TIF)

Table S1 **Population abbreviation (Code), number of sampled individuals per population (n), geographic coordinates (WGS84), altitude, area of grassland patch [m2], percentage of polymorphic loci (PPL), expected heterozygosity (He), average pairwise FST per population (FST.overall), average inbreeding coefficient per population (fAFLP), and average relatedness coefficient per population (rc) for all sampled populations of Briza media, Trifolium montanum and Ranunculus bulbosus.**
(DOCX)

Text S1 **AFLP genotyping protocol.**
(DOCX)
