# Supplementary figures and images for: Patterns of Genetic Variation across Altitude in Three Plant Species of Semi-Dry Grasslands
Source: PLoS One. 2012 Aug 1;7(8):e41608. doi: 10.1371/journal.pone.0041608 (PMC3411590; doi:10.1371/journal.pone.0041608)

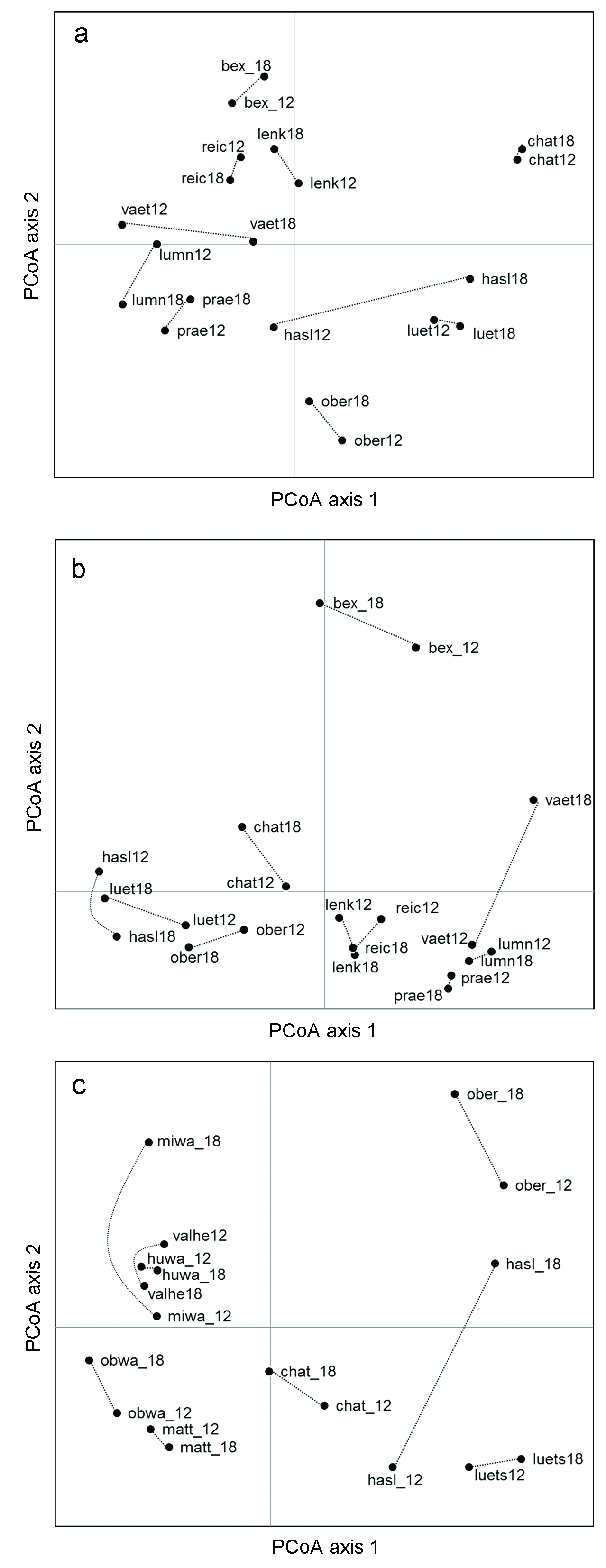

Supplement: Figure S1 — PCoA plots of Nei’s Distances among populations in a) Briza media , b) Trifolium montanum and c) Ranunculus bulbosus . Populations within the same location are connected by dashed lines. (TIF) [file pone.0041608.s001.tif]

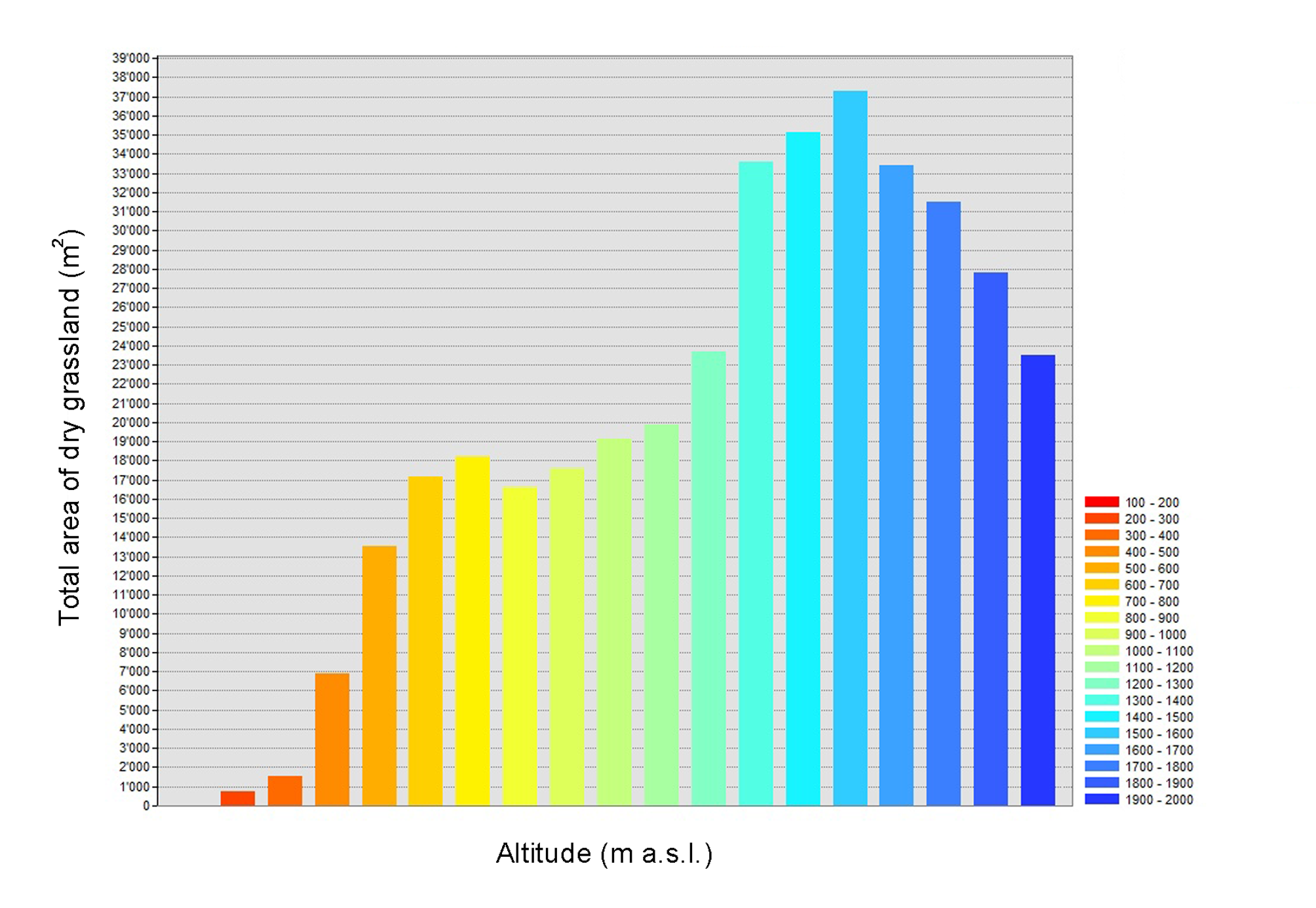

Supplement: Figure S2 — Estimates of total area [m2] of semi dry grasslands between 100 and 2000 m a.s.l. in Switzerland, based on the Swiss national inventory of dry grasslands (TWW). (TIF) [file pone.0041608.s002.tif]

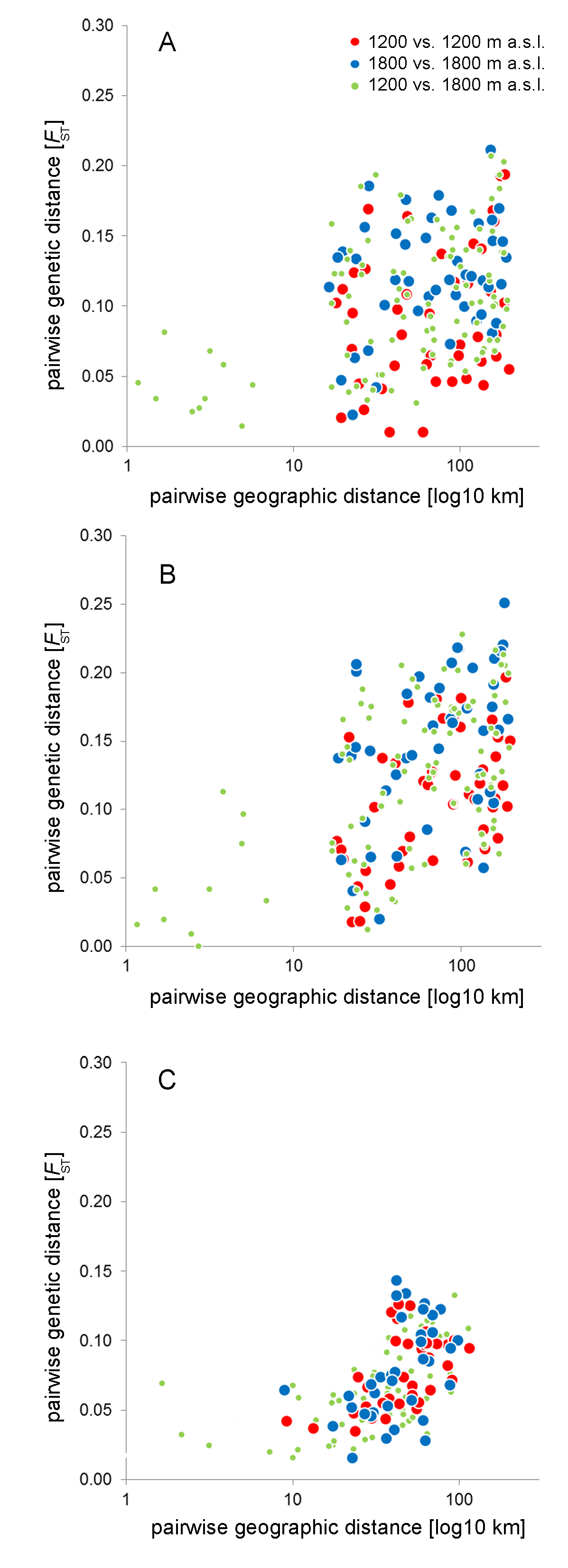

Supplement: Figure S3 — Isolation by distance relationships between pairwise genetic ( F ST) and geographic distances among populations at the same altitudinal level (red = at 1200 m a.s.l.; blue = at 1800 m a.s.l.) and among different altitudinal levels (green = among 1200 and 1800 m a.s.l.) in A) Briza media , B) Trifolium montanum and C) Ranunculus bulbosus . (TIF) [file pone.0041608.s003.tif]
