# Supplementary material for: Patterns of Genetic Variation across Altitude in Three Plant Species of Semi-Dry Grasslands
Source: PLoS One. 2012 Aug 1;7(8):e41608. doi: 10.1371/journal.pone.0041608 (PMC3411590; doi:10.1371/journal.pone.0041608)
Supplement: Table S1 — Population abbreviation (Code), number of sampled individuals per population (n), geographic coordinates (WGS84), altitude, area of grassland patch [m2], percentage of polymorphic loci (PPL), expected heterozygosity (He), average pairwise FST per population (FST.overall), average inbreeding coefficient per population (fAFLP), and average relatedness coefficient per population (rc) for all sampled populations of Briza media, Trifolium montanum and Ranunculus bulbosus. (DOCX) [file pone.0041608.s004.docx]

Table S1: Population abbreviation (Code), number of sampled individuals per population (n), geographic coordinates (WGS84), altitude, area of grassland patch [m^2^], percentage of polymorphic loci (PPL), expected heterozygosity (*H*_e_), average pairwise *F*_ST_ per population (*F*_ST.overall_), average inbreeding coefficient per population (*f*_AFLP_), and average relatedness coefficient per population (rc) for all sampled populations of *Briza media*, *Trifolium montanum* and *Ranunculus bulbosus*.

| Species | Code | n | Long | Lat | Altitude [m a.s.l.] | Patch area | PPL | *H*_e_ | *F*_ST_overall_ | fAFLP | rc |
| --- | --- | --- | --- | --- | --- | --- | --- | --- | --- | --- | --- |
| *Briza. media* | bex_12 | 24 | 7.0907 | 46.2803 | 1166 | 16092 | 85.1 | 0.26 | 0.09 | 0.204 | 0.15 |
|  | bex_18 | 25 | 7.1514 | 46.2961 | 1796 | 70092 | 81.8 | 0.26 | 0.11 | 0.276 | 0.24 |
|  | chat12 | 27 | 7.1303 | 46.4863 | 1258 | 59646 | 85.1 | 0.26 | 0.13 | 0.424 | 0.27 |
|  | chat18 | 28 | 7.1463 | 46.5102 | 1800 | 36773 | 86.8 | 0.27 | 0.13 | 0.502 | 0.31 |
|  | hasl12 | 30 | 8.2272 | 46.7198 | 1176 | 10035 | 89.3 | 0.28 | 0.07 | -0.059 | 0.08 |
|  | hasl18 | 28 | 8.2457 | 46.7286 | 1785 | 108006 | 86.8 | 0.27 | 0.1 | 0.162 | 0.21 |
|  | lenk12 | 28 | 7.4237 | 46.4779 | 1160 | 35727 | 82.6 | 0.28 | 0.07 | 0.192 | 0.08 |
|  | lenk18 | 29 | 7.3623 | 46.5085 | 1814 | 285483 | 81.8 | 0.27 | 0.09 | 0.216 | 0.15 |
|  | luet12 | 30 | 7.9564 | 46.6445 | 1294 | 24045 | 86 | 0.26 | 0.1 | 0.101 | 0.16 |
|  | luet18 | 29 | 7.9716 | 46.6519 | 1754 | 53162 | 86 | 0.25 | 0.11 | 0.118 | 0.21 |
|  | lumn12 | 29 | 9.1633 | 46.7004 | 1157 | 12579 | 86.8 | 0.26 | 0.08 | -0.003 | 0.11 |
|  | lumn18 | 27 | 9.1319 | 46.7042 | 1761 | 23357 | 86 | 0.26 | 0.11 | -0.036 | 0.21 |
|  | ober12 | 29 | 7.4017 | 46.6558 | 1141 | 23987 | 88.4 | 0.26 | 0.12 | -0.081 | 0.23 |
|  | ober18 | 30 | 7.4319 | 46.6767 | 1802 | 175218 | 54.5 | 0.23 | 0.13 | -0.037 | 0.25 |
|  | prae12 | 30 | 9.4056 | 46.7496 | 1185 | 12975 | 87.6 | 0.25 | 0.09 | -0.026 | 0.13 |
|  | prae18 | 28 | 9.3815 | 46.7315 | 1796 | 33959 | 81.8 | 0.25 | 0.09 | 0.017 | 0.13 |
|  | reic12 | 30 | 7.7387 | 46.5797 | 1209 | 8478 | 83.5 | 0.28 | 0.07 | 0.140 | 0.07 |
|  | reic18 | 27 | 7.7536 | 46.5826 | 1679 | 9573 | 82.6 | 0.27 | 0.09 | 0.127 | 0.14 |
|  | vaet12 | 28 | 9.4677 | 46.9679 | 1196 | 23797 | 83.5 | 0.25 | 0.09 | -0.002 | 0.15 |
|  | vaet18 | 29 | 9.4498 | 46.9361 | 1832 | 27676 | 81 | 0.25 | 0.1 | 0.093 | 0.2 |
|  |  |  |  |  |  |  |  |  |  |  |  |
| *Trifolium montanum* | bex_12 | 30 | 7.0907 | 46.2803 | 1167 | 16092 | 58.6 | 0.25 | 0.16 | 0.330 | 0.47 |
|  | bex_18 | 30 | 7.1514 | 46.2961 | 1794 | 70092 | 55.6 | 0.24 | 0.18 | 0.207 | 0.53 |
|  | chat12 | 30 | 7.1102 | 46.4721 | 1208 | 59646 | 51.5 | 0.22 | 0.1 | 0.089 | 0.13 |
|  | chat18 | 29 | 7.1463 | 46.5102 | 1799 | 36773 | 50.5 | 0.21 | 0.16 | 0.340 | 0.38 |
|  | hasl12 | 27 | 8.2272 | 46.7198 | 1176 | 10035 | 86.9 | 0.25 | 0.12 | 0.293 | 0.24 |
|  | hasl18 | 26 | 8.2457 | 46.7286 | 1782 | 108006 | 51.5 | 0.22 | 0.13 | 0.130 | 0.3 |
|  | lenk12 | 30 | 7.4237 | 46.4779 | 1161 | 35727 | 59.6 | 0.25 | 0.09 | -0.002 | 0.13 |
|  | lenk18 | 28 | 7.4016 | 46.418 | 1859 | 24557 | 47.5 | 0.21 | 0.09 | -0.005 | 0.1 |
|  | luet12 | 28 | 7.9564 | 46.6445 | 1294 | 24045 | 55.6 | 0.24 | 0.09 | 0.045 | 0.11 |
|  | luet18 | 30 | 7.9716 | 46.6519 | 1754 | 53162 | 61.6 | 0.25 | 0.13 | 0.205 | 0.23 |
|  | lumn12 | 28 | 9.1633 | 46.7004 | 1157 | 12579 | 54.5 | 0.23 | 0.11 | 0.056 | 0.21 |
|  | lumn18 | 30 | 9.1319 | 46.7042 | 1760 | 23357 | 57.6 | 0.24 | 0.1 | 0.133 | 0.16 |
|  | ober12 | 29 | 7.4017 | 46.6558 | 1145 | 23987 | 55.6 | 0.23 | 0.11 | -0.028 | 0.17 |
|  | ober18 | 29 | 7.4319 | 46.6767 | 1802 | 175218 | 61.6 | 0.25 | 0.1 | -0.031 | 0.16 |
|  | prae12 | 29 | 9.4056 | 46.7496 | 1185 | 12975 | 46.5 | 0.21 | 0.12 | -0.018 | 0.2 |
|  | prae18 | 29 | 9.3815 | 46.7315 | 1797 | 33959 | 49.5 | 0.22 | 0.12 | -0.042 | 0.23 |
|  | reic12 | 30 | 7.7387 | 46.5797 | 1209 | 8478 | 60.6 | 0.25 | 0.09 | 0.101 | 0.1 |
|  | reic18 | 30 | 7.7536 | 46.5826 | 1682 | 9573 | 54.5 | 0.23 | 0.09 | -0.017 | 0.13 |
|  | vaet12 | 29 | 9.4677 | 46.9679 | 1198 | 23797 | 53.5 | 0.23 | 0.1 | 0.065 | 0.14 |
|  | vaet18 | 27 | 9.4498 | 46.9361 | 1832 | 27676 | 90.9 | 0.28 | 0.16 | 0.495 | 0.37 |
|  |  |  |  |  |  |  |  |  |  |  |  |
| *Ranunculus bulbosus* | chat12 | 30 | 7.1303 | 46.4863 | 1259 | 14639 | 57.1 | 0.17 | 0.06 | -0.206 | 0.1 |
|  | chat18 | 31 | 7.1773 | 46.4295 | 1743 | 41742 | 65.1 | 0.18 | 0.05 | -0.035 | 0.08 |
|  | hasl12 | 29 | 8.2639 | 46.7214 | 1151 | 38795 | 43.7 | 0.15 | 0.08 | -0.028 | 0.17 |
|  | hasl18 | 29 | 8.2436 | 46.7278 | 1709 | 108006 | 56.3 | 0.17 | 0.08 | 0.136 | 0.17 |
|  | huwa12 | 29 | 7.1692 | 46.0157 | 1299 | 96030 | 61.1 | 0.18 | 0.06 | 0.291 | 0.09 |
|  | huwa18 | 30 | 7.2607 | 46.1611 | 1733 | 57507 | 59.5 | 0.18 | 0.07 | 0.205 | 0.13 |
|  | luet12 | 28 | 7.9737 | 46.6449 | 1216 | 6332 | 54.8 | 0.16 | 0.08 | -0.128 | 0.15 |
|  | luet18 | 29 | 7.9716 | 46.6519 | 1732 | 58486 | 55.6 | 0.17 | 0.09 | -0.147 | 0.2 |
|  | matt12 | 28 | 7.7971 | 46.2916 | 1122 | 19188 | 49.2 | 0.15 | 0.06 | -0.025 | 0.11 |
|  | matt18 | 28 | 7.923 | 46.2492 | 1854 | 19244 | 48.4 | 0.14 | 0.07 | -0.044 | 0.1 |
|  | miwa12 | 26 | 7.5558 | 46.3295 | 1214 | 18735 | 48.4 | 0.15 | 0.06 | 0.237 | 0.11 |
|  | miwa18 | 26 | 7.326 | 46.309 | 1832 | 115772 | 52.4 | 0.18 | 0.07 | 0.587 | 0.09 |
|  | ober12 | 30 | 7.4036 | 46.6552 | 1099 | 23987 | 55.6 | 0.18 | 0.09 | 0.173 | 0.19 |
|  | ober18 | 29 | 7.4305 | 46.6744 | 1674 | 175218 | 54.8 | 0.18 | 0.08 | 0.487 | 0.14 |
|  | obwa12 | 30 | 7.6853 | 46.3195 | 1230 | 8464 | 56.3 | 0.17 | 0.07 | -0.046 | 0.15 |
|  | obwa18 | 29 | 7.6712 | 46.3362 | 1805 | 29685 | 52.4 | 0.18 | 0.08 | 0.131 | 0.15 |
|  | valhe12 | 30 | 7.4255 | 46.2119 | 1182 | 44231 | 59.5 | 0.18 | 0.06 | 0.195 | 0.09 |
|  | valhe18 | 21 | 7.4828 | 46.131 | 1774 | 26769 | 56.3 | 0.17 | 0.05 | 0.198 | 0.07 |
