# Supplementary material for: Patterns of Genetic Variation across Altitude in Three Plant Species of Semi-Dry Grasslands
Source: PLoS One. 2012 Aug 1;7(8):e41608. doi: 10.1371/journal.pone.0041608 (PMC3411590; doi:10.1371/journal.pone.0041608)
Supplement: Text S1 — AFLP genotyping protocol. (DOCX) [file pone.0041608.s005.docx]

# Text S1

## AFLP genotyping protocol

DNA was extracted from 10 mg of leaf tissue with the DNeasy 96 Plant Kit (Qiagen, Hilden, Germany) following the protocol for lyophilized plant leaf tissue. For increased DNA output, 2 x 50 ul of elution buffer AE was used. The quantity of DNA was measured spectrophotometrically with an 8-capillary NanoDrop 8000 (Thermo Scientific Waltham, Massachusetts). AFLP analyses followed a modified protocol of Vos and colleagues [68] for plants. Appr. 200 ng template DNA each was treated with restriction enzymes in a reaction volume of 40 ul containing 4 ul NEB2-buffer (NEB New England Biolabs, Ipswich, Massachusetts) , 40 ug BSA (NEB), 2 U EcoRI (NEB) and 1 U MseI (NEB) for 1 h at 37°C. For ligation of AFLP adapters, 2.4 ul NEB2-buffer (NEB), 1.2 ug BSA (NEB), 57.6 nmol ATP (NEB), 4.15 pmol each of EcoRI- and MseI-adapters (Microsynth, Balgach, Switzerland) and 23 U T4-Ligase (NEB) was added, increasing the reaction volume to 54 ul. Ligation was for 3 h at room temperature, enzymes were inactivated for 10 min at 65°C. Pre-selective amplification was carried out in 22 ul reaction volumes including 2 ul of restriction/ligation product, 4.2 ul Go Taq-Flexi buffer (Promega, Madison, Wisconsin), 8 nmol dNTPs (Promega), 55.3 nmol MgCl_2_ (Promega), 32 pmol *Mse*I- and *EcoR*I preselective primers (Microsynth) each and 0.5 U Go Taq Polymerase (Promega). Thermo cycling started at 70°C (2min) followed by 20 cycles of 94°C (20sec), 56°C (30 sec) and 72°C (2min) and a final step of 60°C (30 min). Selective amplification was carried out in 12.7 ul reaction volumes including 2.7 ul of 1:10-dilution of pre-selective amplification product, 2.7 ul Go Taq-Flexi buffer (Promega), 5 nmol dNTPs (Promega), 34.1 nmol MgCl_2_ (Promega), 2.2 pmol fluorescence-labled EcoRI primers (ABI), 3.1 pmol MseI primers (Microsynth) and 0.5 U Go Taq Polymerase (Promega). Thermo cycling started at 94°C (2min) followed by 10 cycles of 94°C (20sec), 66°C (30 sec) and 72°C (2min) with 1°C decrease in annealing temperature after each cycle. Subsequently 20 cycles of 94°C (20sec), 56°C (30 sec) and 72°C (2min) were carried out followed by a final step of 60°C (30 min). Quantity of DNA in Restriction/Ligation and pre-selective PCR products were tested on 1.6 % agarose gels.

For AFLP fragment analysis, 3 µl of each of four differently labelled selective PCR-products was added to 10 ul formamide (Applied Biosystems, Foster City, California) and 0.2 µl LIZ 500 size standard (Applied Biosystems). Samples were denatured at 92 °C for three minutes and immediately put on ice. Fragment analyses were performed on an automated capillary sequencer 3730xl DNA Analyzer (Applied Biosystems). Reproducibility of AFLP peaks was tested with double extraction and AFLP analyses of 7 %, 6 % and 5 % of samples in *B. media*, *T. montanum* and *R. bulbosus* respectively.
